# Pleural Fluid GSDMD Is a Novel Biomarker for the Early Differential Diagnosis of Pleural Effusion

**DOI:** 10.3389/fmicb.2021.620322

**Published:** 2021-06-07

**Authors:** Pu Li, Jing Shi, Lijing Zhou, Bo Wang, Li Jun Zhang, Liang Duan, Qin Hu, Xiaolan Zhou, Yuan Yuan, Dandan Li, Hong Chen, Qing Zhao, Xuemei Peng, Weixian Chen

**Affiliations:** ^1^Department of Laboratory Medicine, The Second Hospital of Chongqing Medical University, Chongqing, China; ^2^Department of Laboratory Medicine, The First Hospital of Chongqing Medical University, Chongqing, China; ^3^Department of Medical Record Management, The Second Hospital of Chongqing Medical University, Chongqing, China; ^4^Laboratory Medical College, Chongqing Medical University, Chongqing, China; ^5^Department of Neurology, Chongqing People’s Hospital, Chongqing, China

**Keywords:** GSDMD, pyroptosis, pleural effusion, nucleated cells, biomarker

## Abstract

**Objective:**

Gasdermin D (GSDMD), controlling pyroptosis in cells, has multiple physiological functions. The diagnostic role of GSDMD in pleural effusion (PE) remains unknown.

**Methods:**

Sandwich ELISA kits that we developed were applied to measure the level of GSDMD for 335 patients with a definite cause of PE, including transudative PE, tuberculous pleural effusion (TPE), parapneumonic pleural effusion (PPE), and malignant pleural effusion (MPE). The diagnostic accuracy of Light’s criteria vs. the new marker GSDMD was performed. Clinical follow-up of 40 cases of PPE was conducted and divided into efficacy and non-efficacy groups according to the therapeutic outcome. Nucleated cells (NCs) in PE were isolated and further infected with bacteria to verify the cell source of GSDMD.

**Results:**

The diagnostic accuracy of GSDMD for the diagnosis of PE were 96% (sensitivity) and 94% (specificity). The receiver operating characteristic (ROC) curve indicated that GSDMD can be an efficient biomarker for the differential diagnosis of transudative PE and other groups (all AUC > 0.973). Noteworthily, the highest AUC belonged to tuberculosis diagnosis of 0.990, and the cut-off value was 18.40 ng/mL. Moreover, the same cut-off value of PPE and MPE was 9.35 ng/mL. The combination of GSDMD, adenosine deaminase (ADA), and lactate dehydrogenase (LDH) will further improve the diagnostic efficiency especially between TPE and PPE (AUC = 0.968). The AUC of GSDMD change at day 4, which could predict the therapeutic effect at an early stage, was 0.945 (*P* < 0.0001). Interestingly, bacterial infection experiments further confirm that the pleural fluid GSDMD was expressed and secreted mainly by the NCs.

**Conclusion:**

GSDMD and its combination are candidates as a potentially novel biomarker not only to separate PEs early and effectively, but also monitor disease progression.

## Introduction

Pleural effusion (PE) occurs frequently in respiratory medicine patients. PE accumulation develops when pathological processes result in an imbalance between fluid formation and absorption, including systemic conditions, lung diseases, and organic dysfunction and so on. Tuberculous pneumonia, lung infection, and malignant-pulmonary diseases are the most common causes of PE (Feller-Kopman and Light, 2018).

The formation of an exudate usually implies pleural disease, and the biochemical analysis of PE is widely employed in clinical samples to distinguish the cause of PE. Considered as the golden standard, the microscopic examination of *Mycobacterium tuberculosis* (Mtb) has a limited sensitivity, and the culture of Mtb in the pleural fluid has to be prolonged for several weeks and requires higher laboratory conditions. Lactate dehydrogenase (LDH), measured in the pleural fluid, is implicated in parapneumonic effusion, tuberculous pleuritis, or malignant effusion, thus, it is less specific and sensitive in distinguishing the cause of PE (Lee et al., 2017). Measurement of adenosine deaminase (ADA) will contribute to the diagnosis of tuberculous pleural effusion (TPE). With 63 studies, a meta-analysis of the diagnostic capacity about ADA in tuberculous pleurisy showed that the mean sensitivity and specificity were 0.92 and 0.90, respectively (Liang et al., 2008). Despite the stable cut-off value and well-established standard method and operating protocol of ADA, a recent study found that it can be an independent predictor of a worse survival in patients with malignant pleural effusion (MPE). A higher or lower ADA level is correlated with a worse survival when compared to a normal level, indicating that the level of ADA may not have an obvious diagnostic performance in the differential diagnosis of TPE and MPE, although it passes a great diagnostic efficiency of pleural tuberculosis (Wang et al., 2017). In addition, criticism about the diagnostic performance of ADA in a low prevalence setting has been made (Wang et al., 2017). More cost-effective, high-performance, and method-feasible biomarkers should be explored.

Pyroptosis is a form of inflammatory programmed cell death pathway (Man et al., 2017) activated by caspase-1 or caspase-11/4/5, which cleave gasdermin D (GSDMD) to generate a gasdermin-N domain (NT-GSDMD) and induce the activation and secretion of the two prominent pro-inflammatory cytokines, interleukin-1β (IL-1β) and IL-18 (Jorgensen and Miao, 2015). GSDMD, a 480-amino acid protein, consists of a C-terminal inhibition domain and an N-terminal pore-forming domain which eventually oligomerizes and perforates the plasma membrane that drives pyroptosis (Kovacs and Miao, 2017). There exists a cleavage site between the two domains, D276 of mice and D275 of humans (Broz and Dixit, 2016). Studies have shown that, in non-small cell lung cancer (NSCLC), pneumonia, and pulmonary tuberculosis, the GSDMD level was significantly upregulated (Cheng et al., 2017; Gao et al., 2018; Gong et al., 2019).

Quantitative detection of GSDMD in PE has not been reported yet, and so far, it remains elusive about the relationship between the levels of GSDMD and PE-related diseases. In this study, we made efforts to evaluate the value of GSDMD as a novel parameter to the differential diagnosis of pleural fluid, and get a better insight into the relationship between the levels of pleural fluid GSDMD and the count of Nucleated cells (NCs).

## Materials and Methods

### Study Population

**FIGURE 1:**
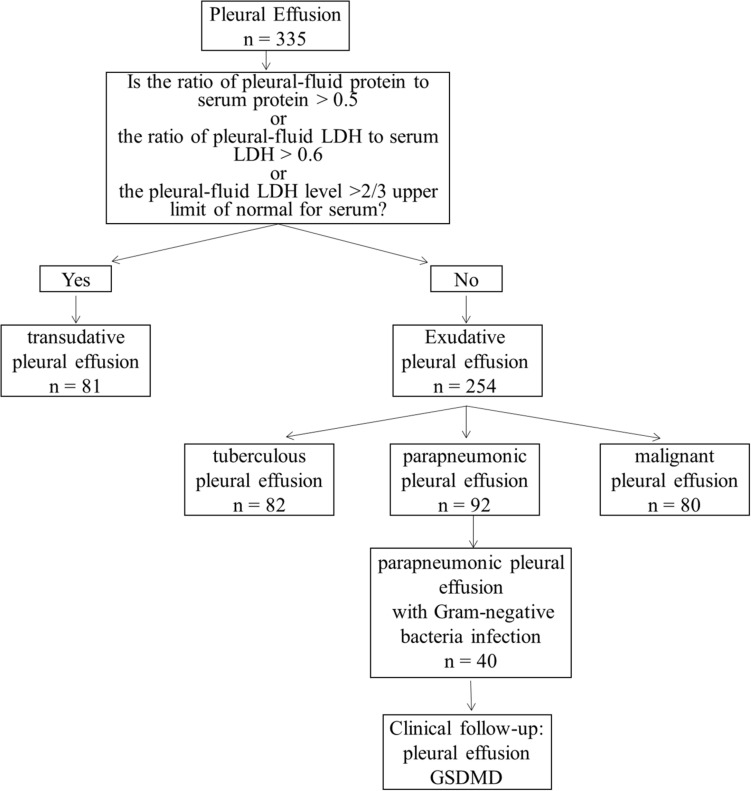
Flow chart of the study groups.

Admitted to the Second Affiliated Hospital of Chongqing Medical University between October 2017 and November 2019, a total of 335 samples collected from clinical patients in respiratory medicine were divided into four groups, including 81 cases of transudative PE (the control group), 82 cases of TPE, 80 cases of MPE, and 92 cases of parapneumonic pleural effusion (PPE). All patients did not place the drainage tube, and the first thoracentesis pleural fluid sample was collected. Clinical follow-up of 40 cases of PPE with Gram-negative bacterial infection would have been done to define if the change of PE-GSDMD might provide information about the efficacy evaluation of pneumonia (Figure 1). TPE and MPE samples were obtained from tuberculous pleurisy and NSCLC patients, respectively. Tuberculous pleurisy was diagnosed when one of the following criteria was met: (1) positive culture for Mtb in the pleural fluid, pleural tissue, sputum, or bronchial aspirate; (2) pathological finding of chronic granulomatous pleural inflammation without evidence of other granulomatous diseases (Light, 2010). Patients with NSCLC are confirmed by a histopathology analysis of the pleural biopsy, or/and cytologic examination of the PE in terms of the diagnosis and treatment guide issued by the National Comprehensive Cancer Network (NCCN). Patients coupling with lung cancer and tuberculous pneumonia were excluded from this study. The PPE patients were based on the criteria: (1) exudates were linked with bacterial pneumonia, lung abscesses, or bronchiectasis; (2) people who manifest as inflammatory pleuritis, chronic empyema, or pleural fibrosis and plaques, without Mtb observed in the pleural fluid. Transudative PE was confirmed based on Light’s criteria (Light et al., 1972). PEs with all of the following criteria were classified as transudative: (1) the ratio of pleural fluid to serum protein is less than 0.5; (2) the ratio of pleural fluid to serum LDH is less than 0.6; and (3) pleural-fluid LDH is less than 2/3 the normal upper limit. When the pleural fluid definitions of Light’s criteria and clinical appearance were opposite, transudative effusion could also be classified if the serum albumin level minus pleural-fluid albumin level is more than 1.2 g/dl (Burgess et al., 1995).

This study was performed according to the principles of the Declaration of Helsinki and was approved by the Medical Ethics Committee of the Second Affiliated Hospital of Chongqing Medical University. The clinical characteristics of the subject in the study were presented in Table 1.

**TABLE 1:** Biochemical and cytological characteristics in pleural effusions*.

	TPE	MPE	PPE	Control
Patients, no.	82	80	92	81
Males/females	42/40	43/37	49/43	45/36
Age (years)	50.50 ± 15.69	61.55 ± 10.67^†‡^	53.50 ± 13.72	53.81 ± 14.52
GSDMD (ng/mL)	49.38 ± 21.17^†‡^	27.96 ± 14.17^‡^	26.25 ± 13.37^‡^	4.70 ± 3.52^†^
LDH (U/L)	378.97 ± 287.73^‡^	393.13 ± 319.35^‡^	181.47 ± 82.79	134.85 ± 121.96
ADA (U/L)	37.09 ± 15.91^†‡^	21.88 ± 12.17^†‡^	14.25 ± 6.60^†‡^	8.06 ± 4.81^†‡^
Nucleated cell counts, *10^6^/L	4,078.55 ± 2,842.70^‡^	3,863.00 ± 3,183.23^‡^	942.98 ± 480.23^†‡^	101.26 ± 53.94^†‡^

LDH: lactate dehydrogenase; ADA: adenosine deaminase; TPE: tuberculous pleural effusion; MPE: malignant pleural effusion; PPE: parapneumonic pleural effusion; *Values are presented as mean ± SD. ^†^*p* < 0.01 compared with each of the other three groups. ^‡^*p* < 0.01 compared with the control group. The comparisons were determined by the one-way analysis of variance or Chi-square test.

### Quantitative Analysis of Pleural Fluid ADA, LDH, and NC Count

Pleural effusion was collected according to the standard operating protocol and was immediately transferred to the clinical laboratory. Pleural fluid was then centrifuged at 1,500 rpm for 10 min at room temperature, and supernatants were collected and stored at −80°C for analysis later. IFN-γ levels were detected by an ELISA kit (WANTAI BioPharm, Beijing, China) following the manufacturer’s protocols. The activity of ADA and LDH was detected by the Hitachi Modular 7600 chemistry analyzer (Hitachi, Tokyo, Japan) with the test kit (Maccura, Sichuan, China). IL-1β was detected by the IMMULITE ^®^ 1000 chemiluminescence immunoassay analyzer (Siemens, Germany). The NCs count in the pleural fluid was quantitatively analyzed by the Sysmex XT4000i Automatic Blood Analyzer (Sysmex, Kobe, Japan). Finally, we also performed Mtb gene testing on TPE according to the manufacturer’s instructions.

### Nucleated Cells Isolate and Cell Infection

Nucleated cells were isolated aseptically using the Ficoll-Hypaque gradient centrifugation method from PE and cultured in RPMI-1640 supplement in 6-well plates (10^6^ cells/well). In our experiments, *Escherichia coli* (*E. coli*), *Staphylococcus aureus* (*S. aureus*), *Salmonella*, and *Pseudomonas aeruginosa* (*P. aeruginosa*) were added into the culture, respectively (bacterium: cell = 30:1). At various time points of 0 (control), 1, 2, 4, 8, 16, 24, and 48 h, supernatants were harvested for determining the GSDMD concentrations. At the same time, the intracellular GSDMD proteins at the various time points of 0 (Control), 1, 2, 4, and 8 h were determined. In addition, we also detected the concentration of LDH, IL-1β, and IFN-γ at time points of 0 (Control), 1, 2, 4, and 8 h in the culture supernatants at the presence of *E. coli*, *S. aureus*, *P. aeruginosa*, and *Salmonella*.

### PE-GSDMD in Patients Who Would Have a Response or Stable Disease to Antibiotic

This was a non-intervention study. The suspected infected patients were given an empirical anti-infection treatment for the first time. The drug selection was based on the guidelines (Kalil et al., 2016) and epidemiology, and was adjusted according to the clinical response and/or drug sensitivity after the initial efficacy evaluation. The course of treatment was combined with the guidelines (Kalil et al., 2016) and treatment response. GSDMD samples were collected in the baseline and days 2 and 4 in the duration of the therapy. The efficacy evaluation was assessed and divided into efficacy and non-efficacy at the end of treatment by a group of three or more doctors according to the guiding principle of clinical trials on anti-bacterial drugs by the Ministry of Health (Antibacterial Drug Clinical Trials Technical Guidelines’ Writing Group, 2014). Clinical efficacy, microbiological efficacy, and the comprehensive curative effect were included in the evaluation criteria but without GSDMD.

### Analysis of Pleural Fluid GSDMD

Gasdermin D was measured by an ELISA method as we previously described (Xiong et al., 2018). In short, 100 μL of serum samples or standard (recombinant Human GSDMD protein; Abcam, United States) were added into each capture antibody-coated well (anti-GSDMD antibody; Abcam, United States) and incubated for 1 h at 37°C. After aspirating and washing each well three times, 100 μL of diluted detection antibody-conjugated HRP (rabbit anti-human IgG H&G antibody; Abcam, United States) was added and incubated for 30 min at 37°C. Repeating the aspiration/wash of each well three times, 100 μL of substrate solution was added and incubated at room temperature for 30 min. Next, 50 μL of stop solution (1 mol/L H_2_SO_4_) was added to each well. The absorbance of the colored solution of GSDMD was measured at 450 nm by using a MultiskanTM FC microplate reader (Thermo Fisher Scientific, Waltham, MA, United States).

### Statistical Analysis

All statistical analysis was performed with the SPSS software, version 16.0 (SPSS, Inc., Chicago, IL, United States). The clinical characteristics of patients were presented as means ± SD. Parametric tests were used since the data were normally distributed as determined by a normality test. Comparison of continuous variables was made by the Student’s *t*-test and Spearman’s correlation. Differences among more than two groups were compared by the one-way analysis of variance (one-way ANOVA). All tests in this study were two tails and a *P*-value < 0.05 was considered statistically significant. Receiver operating characteristic (ROC) curves were used to evaluate the diagnostic accuracy of GSDMD, LDH, ADA, and the combination of the three biomarkers. Sensitivity, specificity, and the areas under ROC curves (AUC) were included. The Light’s criteria vs. the new marker GSDMD was further performed to compare the diagnostic accuracy. Youden index was used to identify the best cut-off values for each variable.

## Results

### Correlation Analysis of GSDMD, ADA, and LDH

**FIGURE 2:**
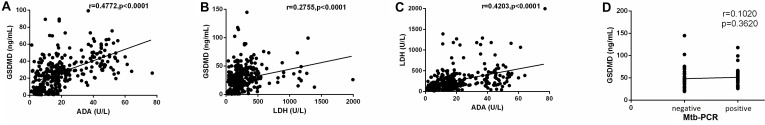
Correlation analysis of GSDMD with ADA **(A)** (*n* = 335), GSDMD with LDH **(B)** (*n* = 335), ADA with LDH **(C)** (*n* = 335), and GSDMD with Mtb-PCR **(D)** (*n* = 82). ADA: adenosine deaminase; LDH: lactate dehydrogenase. Correlations were determined by Pearson correlation coefficients. GSDMD content in PE was positively correlated with ADA (*r* = 0.4772, *p* < 0.0001) and with LDH (*r* = 0.2755) (*p* < 0.0001), which indicated that GSDMD could turn into an effective biomarker for PE differential diagnosis.

As we discussed before, ADA, LDH, and Mtb-PCR biochemical analysis of PE is widely employed in clinical samples to distinguish the cause of PE. In the first part of our experiment, in order to find the correlation of GSDMD, a novel biomarker, and other usual biochemical indexes, we investigated the activity of ADA and LDH and the result of the Mtb-PCR assay in these four groups. Then, we made correlation analyses among the result of these four assays. Interestingly, there was a statistically significant relationship among GSDMD, ADA, and LDH when all patients with pleural exudates were considered. As shown in Figure 2, the results illustrated that the GSDMD content in PE was positively correlated with ADA (*r* = 0.4772, *p* < 0.0001) and with LDH (*r* = 0.2755) (*p* < 0.0001), which indicated that GSDMD could turn into an effective biomarker for PE differential diagnosis.

### Diagnostic Value of GSDMD for PE Differential Diagnosis

**FIGURE 3:**
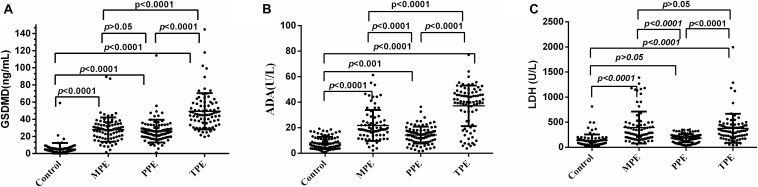
Comparisons of GSDMD **(A)**, ADA **(B)**, and LDH **(C)** concentrations with TPE (*n* = 82), MPE (*n* = 80), PPE (*n* = 93), and transudative pleural effusion (*n* = 81). TPE: tuberculous pleural effusion; MPE: malignant pleural effusion; PPE: parapneumonic pleural effusion; Horizontal bars indicate means. Statistical analysis was done by one-way analysis of variance. The concentration of GSDMD in these four groups (*p* < 0.0001), except between MPE and PPE (*P* > 0.05), but not of ADA and LDH.

**FIGURE 4:**
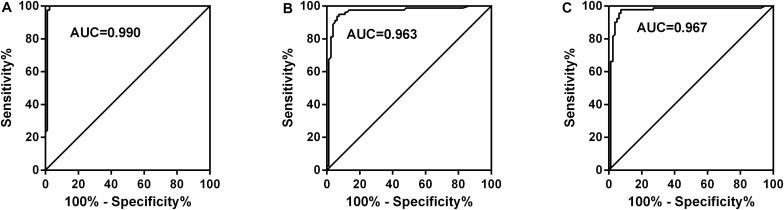
Receiver operating characteristic curves of GSDMD for differential diagnosis of Control (*n* = 81) versus TPE (*n* = 82) **(A)**, Control (*n* = 81) versus MPE (*n* = 80) **(B)**, and Control (*n* = 81) versus PPE (*n* = 92) **(C)**. TPE: tuberculous pleural effusion; MPE: malignant pleural effusion; PPE: parapneumonic pleural effusion; AUC: area under curve. GSDMD showed a pretty high diagnostic capacity for distinguishing transudative PE from TPE (AUC = 0.990), MPE (AUC = 0.963), and PPE (AUC = 0.967).

We analyzed whether or not the level of GSDMD was associated with a different kind of PE. Interestingly, there was a conspicuous statistical significance among the concentration of GSDMD in these four groups (*p* < 0.0001), except between MPE and PPE (*P* > 0.05) (Figure 3A), but not of ADA and LDH (Figures 3B,C). To evaluate the usefulness of a new parameter, GSDMD, for separating transudative PE from exudative PE, and to compare the results with Light’s criteria, the sensitivity, specificity, PPV, NPV, and AUC were calculated (Table 2). Compared with Light’s criteria (sensitivity: 98%; specificity: 83%), the diagnostic accuracy of GSDMD for the diagnosis of PE was 96% (sensitivity) and 94% (specificity). The capacity of GSDMD to differentiate PEs was assessed with the ROC curve analyses (Figure 4 and Table 3). GSDMD showed a pretty high diagnostic capacity for distinguishing the transudative PE from TPE (AUC = 0.990), MPE (AUC = 0.963), and PPE (AUC = 0.967) (Figure 4), while the AUC of ADA was determined as 0.937, 0.896, and 0.771, and the AUC of LDH was determined as 0.872, 0.864, and 0.710, respectively (Table 3). With the highest level of AUC, the cut-off value, sensitivity, and specificity when GSDMD was used to differentiate TPE and transudative PE were 18.40 ng/mL, 100, and 98.77%, respectively. GSDMD can also be an efficient biomarker for the differential diagnosis of PPE and MPE from transudative PE with the same cut-off value of 9.350 ng/mL, indicating the lower diagnostic capacity in the differential diagnosis between PPE and MPE. Additionally, for diagnostic sensitivity alone, compared with ADA and LDH, GSDMD was the highest for differentiating transudative PE from MPE, TPE, and PPE. Furthermore, GSDMD also did well in the differential diagnosis between TPE and PPE (AUC = 0.885) and TPE and MPE (AUC = 0.848).

**TABLE 2:** Diagnostic accuracy for the diagnosis of exudative pleural effusions.

Test	Sensitivity,% (95% CI)	Specificity,% (95% CI)	PPV, % (95% CI)	NPV, % (95% CI)	Area Under curve	*P* value
Light’s criteria (one or more of the following three)	98	83	/	/	/	/
Ratio of pleural-fluid protein level to serum protein level > 0.5	86	84	/	/	/	/
Ratio of pleural-fluid LDH level to serum LDH level > 0.6	90	82	/	/	/	/
Pleural-fluid LDH level > two thirds the upper limit of normal for serum LDH level	82	89	/	/	/	/
GSDMD	96 (93–98)	94 (86–98)	98 (95–99)	89 (80–94)	0.97	<0.0001

*LDH: lactate dehydrogenase; GSDMD: Gasdermin D; PPV, positive predictive value; NPV, negative predictive value.*

**TABLE 3:** The diagnostic accuracy of biomarkers for pleural effusion differential diagnosis.

Group		Control versus TPE	Control versus MPE	Control versus PPE	TPE versus PPE	TPE versus MPE	PPE versus MPE
**Biomarker**							
GSDMD	AUC (*p* value)	0.990 (<0.0001)	0.963 (<0.0001)	0.967 (<0.0001)	0.885 (<0.0001)	0.848 (<0.0001)	0.557 (0.2016)
	Sensitivity, % (95% CI)	100 (94–100)	95.00 (87–98)	97.83 (92–99)	75.61 (67–84)	90.00 (81–95)	45.00 (34–56)
	Specificity, % (95% CI)	98.77 (92–99)	93.83 (86–98)	93.83 (86–98)	88.04 (79–94)	68.29 (57–78)	72.83 (62–81)
	PPV, % (95% CI)	98.80 (93–99)	93.82 (85–98)	94.74 (88–98)	84.93 (74–92)	87.50 (76–94)	59.02 (46–71)
	NPV, % (95% CI)	100 (94–100)	95.00 (87–98)	97.44 (90–99)	80.20 (71–87)	73.47 (63–82)	60.36 (51–69)
	LR+ (95% CI)	81 (12–569)	15.39 (7–36)	15.85 (7–37)	6.32 (4–11)	6.83 (3–13)	1.66 (1–3)
	LR- (95% CI)	0	0.05 (0.02–0.1)	0.02 (0–0.09)	0.28 (0.2–0.4)	0.35 (0.3–0.5)	0.76 (0.6–0.9)
	Youden Index	97.53	87.59	90.42	63.65	58.29	17.13
	Cut-off value (ng/mL)	18.400	9.350	9.350	35.900	39.450	29.150
ADA	AUC (*p* value)	0.937 (<0.0001)	0.896 (<0.0001)	0.771 (<0.0001)	0.863 (<0.0001)	0.758 (<0.0001)	0.713 (<0.0001)
	Sensitivity, % (95% CI)	79.3(68–87)	81.3 (70–89)	83.7 (74–90)	96.7 (90–99)	82.5 (72–90)	70.0 (60–79)
	Specificity, % (95% CI)	100 (95–100)	82.7 (72–90)	63.0 (51–73)	74.4 (63–83)	74.4 (63–83)	84.1 (74–91)
LDH	AUC (*p* value)	0.872 (<0.0001)	0.864 (<0.0001)	0.710 (<0.0001)	0.802 (<0.0001)	0.536 (0.4331)	0.747 (<0.0001)
	Sensitivity, % (95% CI)	78.0 (67–86)	81.3(70–89)	69.6 (59–78)	84.8 (75–91)	57.5 (45–68)	84.8 (75–91)
	Specificity, % (95% CI)	90.1 (81–95)	79.0(68–87)	71.6 (60–81)	65.9 (54–75)	59.8 (48–70)	57.50 (45–68)

*Data are shown as numbers or percentages (95% CI). LDH: lactate dehydrogenase; ADA: adenosine deaminase; TPE: tuberculous pleural effusion; MPE: malignant pleural effusion; PPE: parapneumonic pleural effusion; PPV, positive predictive value; NPV, negative predictive value; LR+: positive likelihood ratio; LR-: negative likelihood ratio. Gasdermin D showed the highest diagnostic capacity for distinguishing transudative PE from TPE (AUC = 0.990), MPE (AUC = 0.963), and PPE (AUC = 0.967).*

### Diagnostic Value of the Combined Detection of GSDMD, ADA, and LDH for PE Differential Diagnosis

In clinical diagnosis, a combination of indexes is generally used to improve the diagnostic efficiency. Like GSDMD, the combination of these three biomarkers displayed an outstanding performance in the differential diagnosis between pleural transudate and TPE, with 100% sensitivity and 97.5% specificity (AUC = 0.998). Moreover, the combination exhibited a higher diagnostic ability for the differential diagnosis between pleural transudate and MPE (AUC = 0.968), pleural transudate and PPE (AUC = 0.973), and TPE and PPE (AUC = 0.968) (Table 4). What is more, there existed a higher diagnostic specificity and sensitivity when using combined indexes than a single index.

**TABLE 4:** Diagnostic value of combined detection of GSDMD, ADA, and LDH for pleural effusion differential diagnosis.

	Control versus TPE	Control versus MPE	Control versus PPE	TPE versus PPE
Area under curve (95% CI)	0.998 (0.995–1.001)	0.968 (0.942–0.995)	0.973 (0.945–1.001)	0.968 (0.942–0.994)
*P* value	<0.0001	<0.0001	<0.0001	<0.0001
Sensitivity, % (95% CI)	100.0 (94–100)	93.75 (86–97)	95.65 (89–99)	87.80 (78–94)
Specificity, % (95% CI)	97.53 (91–100)	96.30 (89–99)	96.30 (89–99)	98.91 (93–100)
PPV, % (95% CI)	97.62 (91–100)	96.15 (88–99)	96.70 (90–99)	98.63 (92–100)
NPV, % (95% CI)	100.0 (94–100)	96.98 (86–98)	95.12 (87–98)	90.10 (82–95)
LR+ (95% CI)	40.5 (10–159)	25.31 (8–77)	25.82 (9–78)	80.78 (11–568)
LR- (95% CI)	0	0.06 (0.03–0.15)	0.05 (0.02–0.12)	0.12 (0.07–0.22)

*Data are shown as numbers or percentages (95% CI: 95% confidence interval). LDH: lactate dehydrogenase; ADA: adenosine deaminase; TPE: tuberculous pleural effusion; MPE: malignant pleural effusion; PPE: parapneumonic pleural effusion; PPV, positive predictive value; NPV, negative predictive value; LR+: positive likelihood ratio; LR-: negative likelihood ratio. The combination exhibited a higher diagnostic ability for differential diagnosis between pleural transudate and MPE (AUC = 0.968), pleural transudate and PPE (AUC = 0.973), and TPE and PPE (AUC = 0.968).*

### Correlation Between PE-GSDMD Activity and Efficacy of PPE Treatment

**FIGURE 5:**
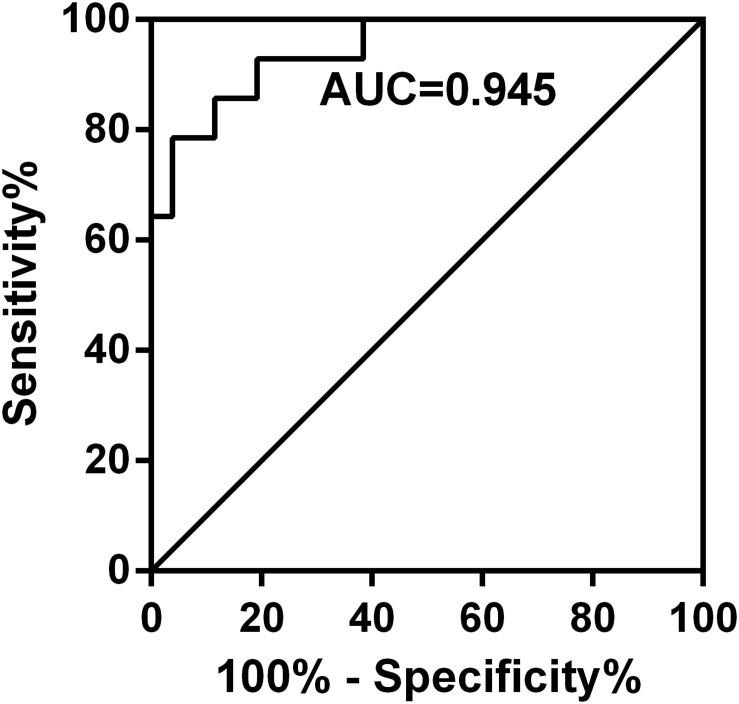
Receiver operating characteristic curves for the percentage change in PE-GSDMD at day 4 in predicting which patients would have a partial response or stable disease. The percentage change in PE-GSDMD at day 4 is anticipated to have a diagnostic accuracy with an AUC of 0.945 (78.57% sensitivity and 96.15% specificity) in predicting whether patients would have a response or stable disease to the antibiotic.

PE-GSDMD of baseline and days 2 and 4 in the duration of therapy were evaluated in all patients in both groups. Mean PE-GSDMD of each point was significantly lower in the efficacy group than the non-efficacy group [baseline: (35.28 ± 18.72) ng/mL versus (26.42 ± 11.09) ng/mL (*p* = 0.1138); day 2 in the duration of therapy (27.72 ± 14.53) ng/mL versus (25.45 ± 7.57) ng/mL (*p* = 0.5898); day 4 in the duration of therapy: (15.25 ± 12.48) ng/mL versus (35.01 ± 14.78) ng/mL (*p* < 0.0001);Table 5]. The level of PE-GSDMD gradually declined from the baseline during the treatment in the efficacy group, while the non-efficacy group presented an uptrend. A 18.82 and 59.15% decrease in PE-GSDMD from the baseline was observed at days 2 and 4 during therapy, respectively (*p* = 0205, *p* < 0.0001, Table 5). In predicting whether patients would have a response or stable disease to the antibiotic, the percentage change in PE-GSDMD at day 4 is anticipated to have a diagnostic accuracy, with an AUC of 0.945 (78.57% sensitivity and 96.15% specificity) (Figure 5).

**TABLE 5:** The trend of GSDMD changes in PPE due to the therapeutic effect.

	The efficacy group (*n* = 26)		The non-efficacy group (*n* = 14)		
		
	Mean (ng/mL)	SD (ng/mL)	Mean (ng/mL)	SD (ng/mL)	*P*
**PE-GSDMD activity**					
Baseline	35.28	18.72	26.42	11.09	0.1138
Day 2	27.72	14.53	25.45	7.57	0.5898
Day 4	15.25	12.48	35.01	14.78	<0.0001
**% of GSDMD change from baseline**					
Day 2	−18.82	19.64	13.67	63.71	0.0205
Day 4	−59.15	25.61	47.13	68.77	<0.0001

*GSDMD: Gasdermin D; PPE: parapneumonic pleural effusion.*

### Correlation Analysis of GSDMD and NCs

**FIGURE 6:**
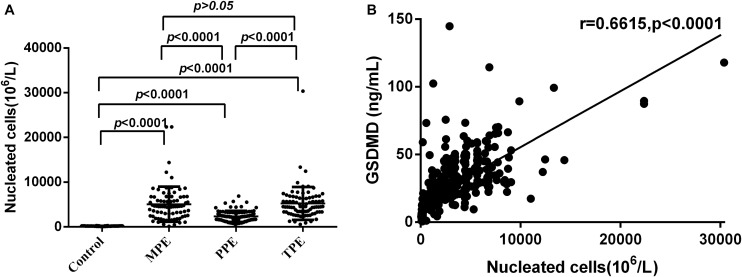
Comparisons of nucleated cells (NCs) count in pleural effusion in the Control (*n* = 81), MPE (*n* = 80), TPE (*n* = 82), and PPE (*n* = 92) groups **(A)** and concentrations of GSDMD correlated with the numbers of NCs in pleural effusion (*n* = 335) **(B)**. TPE: tuberculous pleural effusion; MPE: malignant pleural effusion; PPE: parapneumonic pleural effusion; Horizontal bars indicate means. Statistical analysis was done by one-way analysis of variance. Correlations were determined by Pearson correlation coefficients. There was a significant statistical difference between the pathological groups and PE (Control) (all *P* < 0.0001), except between MPE and TPE (*P* > 0.05), and the concentrations of GSDMD were positively correlated with the numbers of pleural NCs (*r* = 0.6615; *P* < 0.0001), suggesting that pleural fluid GSDMD might be produced mainly by these local NCs.

During the study, we found that the subjects with etiologies showed a notable elevation of total NC counts. As shown in Figure 6A, there was a significant statistical difference between the pathological groups and PE (Control) (all *P* < 0.0001), except between MPE and TPE (*P* > 0.05). Hence, we made a correlation analysis between the level of NCs and GSDMD. As expected, the concentrations of GSDMD in tuberculous, malignant, and parapneumonic PE were all positively correlated with the numbers of pleural NCs (*r* = 0.6615; *P <* 0.0001) (Figure 6B), suggesting that pleural fluid GSDMD might be produced mainly by these local NCs.

### The Results of Cell Infection *in vitro* Culture

**FIGURE 7:**
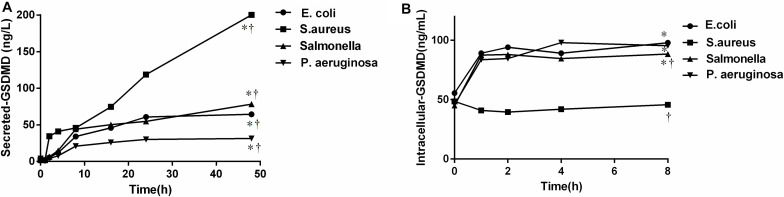
The concentration of the secreted **(A)**/intracellular **(B)**-GSDMD by nucleated cells was cultured, respectively *in vitro* in the presence of bacteria. *E. coil*: *Escherichia coli*; *S. aureus*: *Staphylococcus aureus*; *P. aeruginosa*: *Pseudomonas aeruginosa*. The comparisons were determined by one-way analysis of variance. *p < 0.0001 48 h **(A)** or 8 h **(B)** at each group compared with 0 h; ^†^*p* < 0.05 compared with each of the other three groups at 48 h **(A)** or 8 h **(B)** determined by the one-way analysis of variance. All bacteria were capable of inducing GSDMD production from NCs in a time-dependent manner.

**FIGURE 8:**
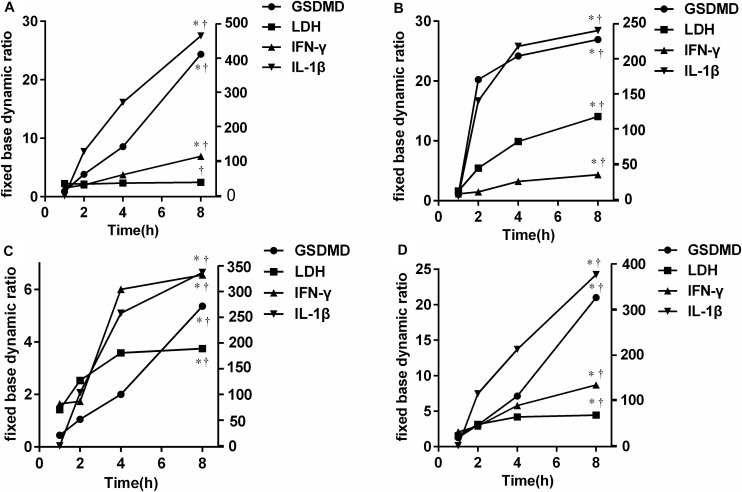
Trend analysis [fixed base dynamic ratio = assay value/fixed value (Control)] of GSDMD, LDH, IFN-γ, and IL-1β in the culture supernatants at the presence of *E. coli* **(A)**, *S. aureus* **(B)**, *P. aeruginosa* **(C)**, and *Salmonella* **(D)**. LDH: lactate dehydrogenase; IFN-γ: interferon-γ; IL-1β: interleukin-1β; *E. coli*: *Escherichia coli*; *S. aureus*: Staphylococcus aureus.*p < 0.0001 8 h at each group compared with 1 h; ^†^*p* < 0.0001 compared with each of the other three groups at 8 h determined by one-way analysis of variance. Compared with LDH and IFN-γ but not with IL-1β, GSDMD displayed superiority both in the time that the biomarker began to grow and the degree of growth at the presence of *E. coli*, *S. aureus*, and *Salmonella*.

To further verify the source of GSDMD, NCs were isolated and cultured *in vitro* in the presence of *E. coli*, *S. aureus*, *P. aeruginosa*, and *Salmonella*, respectively. As shown in Figure 7, all bacteria were capable of inducing GSDMD production from NCs in a time-dependent manner. At the time point of 2 h, the concentration of secreted-GSDMD began increasing significantly, especially in the presence of *S. aureus* (a 20-fold increase compared with the control). As to the level of intracellular GSDMD, after 1 h, there was almost no growth. In Figure 8, we adopted a trend analysis (fixed base dynamic ratio = assay value/fixed value) which adopted the concentration at 0 h as a fixed value to analyze the variation tendency of the concentrations of GSDMD, LDH, IFN-γ, and IL-1β in the culture supernatants at the presence of *E. coli*, *S. aureus*, *P. aeruginosa*, and *Salmonella*. With the extension of the incubation time, the supernatant level of GSDMD, IL-1β, and LDH were significantly elevated after NCs were incubating with the bacteria. What is more, compared with LDH and IFN-γ but not with IL-1β, GSDMD displayed superiority both in the time that the biomarker began to grow and the degree of growth at the presence of *E. coli*, *S. aureus*, and *Salmonella*. Nevertheless, in the presence of *P. aeruginosa*, IFN-γ possessed the maximum fixed base dynamic ratio.

## Discussion

To our knowledge, the current study was the first one to investigate the diagnostic value of GSDMD in the differential diagnosis of different kinds of PE (including pleural transudate, TPE, MPE, and PPE). Our results indicate that the concentration of GSDMD not only acts as a novel biomarker for the differential diagnosis of pleural fluid with a high diagnostic sensitivity and disease progression prediction but can also combine with ADA and LDH to improve its diagnostic ability. Moreover, PE-GSDMD displayed drug efficacy monitoring. Additionally, we found that GSDMD in PE was secreted by NCs, manifested by the correlation analysis and cell infection results.

Pyroptosis is a form of pathogen-induced and caspase-dependent cell death type characterized by the exposure of phosphatidylserine, pore-formation, cell membrane rupture, and secretion of cytoplasmic contents, including IL-1β, LDH, and IL-18 (Ding et al., 2016). Recent studies show that pyroptosis can be triggered by diverse pathological stimuli such as infectious disease, nervous system diseases, and cancer. When the body is infected with bacteria, lipopolysaccharide (LPS) will combine with caspase-4, caspase-5, and caspase-11, acting as cytosolic LPS receptors and induce cell pyroptosis (Shi et al., 2015; Yang et al., 2019). Similarly, through pyroptosis, macrophages infected with Mtb can destroy the survival environment of Mtb, and eventually prevent Mtb replication (Fogel, 2015; Lerner et al., 2015). Mtb activated the canonical NLRP3 inflammasome by inducing potassium efflux upon the infection of monocytes and macrophages, followed by the GSDMD-dependent pyroptosis (Beckwith et al., 2020). NLRP3 and AIM2 are considered two crucial kinds of pattern recognition receptors (PRR) which can activate cell pyroptosis after recognizing the pathogen-associated molecular patterns (PAMP) (Jin and Flavell, 2010; Ma et al., 2016). According to a large number of reports, microRNA induction (Jiang et al., 2017), chemotherapy drugs (Wang et al., 2017a), LXRs receptor mediation (Derangère et al., 2014), and other manners can induce the occurrence of pyroptosis of tumor cells. Thereinto, microRNA, approximately 21–23 nucleotides in length, can directly target caspase-1, thereby inhibiting tumor cell proliferation and migration (Jiang et al., 2017).

A recent report displays that mediation by the ESCRT III components, a calcium-dependent membrane repair machinery, can antagonize the execution phase of pyroptosis (Broz and Dixit, 2016). Calcium flux is an evolutionarily conserved trigger for cell membrane repair through the exocytosis of vesicles such as lysosomes, mobilization of annexins, and recruitment of the ESCRT machinery to the sites of membrane injury (Cooper and McNeil, 2015). Based on this membrane repair machinery, cell-free culture supernatants will detect GSDMD, as what has been reported (Liu et al., 2016). What is more, what could be detected in the culture supernatants is GSDMD-NT, and GSDMD was only detected in the cell. Additionally, pyroptosis is a kind of cell lytic death way. After membrane rupture, GSDMD is released in large quantities. These are the reasons why GSDMD can be detected in PE.

Biochemical testing of body fluid has been widely performed in the differential diagnosis between transudate and pathological PEs. Though traditional biomarkers, such as ADA, LDH, glucose, and protein, have been applied and new markers like IL-27 (Wang et al., 2018), IFN-γ (Tang et al., 2019), CXCL9, and CXCL11 (Chung et al., 2017) have been developed, ADA is still the most cost-effective pleural fluid marker. However, its value is questioned in differential diagnosis and a low prevalence setting. We have previously reported the use of proteome analysis for the differential diagnosis of TPE and MPE (Shi et al., 2019). In this study, the measurement of GSDMD is suggested as a reliable test in the separation of pleural exudates from transudates with an accuracy similar to that of Light’s criteria. We innovatively developed GSDMD, an indispensable molecular requirement in pyroptosis, as a novel biomarker for a PE-related disease diagnosis and the differential diagnosis of PE. GSDMD showed a higher diagnostic accuracy than ADA and LDH in distinguishing transudate and other pathological PEs, with a 0.973 average level of the AUC for GSDMD, 0.868 for ADA, and 0.815 for LDH. It is worth mentioning that the sensitivity and specificity for distinguishing transudate from TPE were 100% and 98.77% (AUC = 0.990) at the cut-off value of 18.40 ng/mL. What is more, GSDMD not only manifests a predominant advantage in the differential diagnosis of TPE but also performs well in the differential diagnosis between TPE and PPE (AUC = 0.885), and TPE and MPE (AUC = 0.848). To make a better clinical practical performance, we also calculated the cut-off value for GSDMD, which was selected based on the highest diagnostic accuracy. Although there existed some deficiency of GSDMD in the differential diagnosis of PE, such as the differential diagnosis between MPE and PPE, and TPE and PPE, it was indisputable that GSDMD had a good diagnostic performance. Furthermore, the combination of the three indexes further improved the diagnostic ability.

As we mentioned before, GSDMD can be detected in PE as a sign of pyroptosis, in theory. Interestingly, there existed an arresting positive correlation between the concentration of GSDMD and the count of NCs, which suggested that GSDMD might be secreted by these pleural local NCs. After the NCs were infected by bacteria, the level of GSDMD in the culture supernatants presented a growth trend in a time-dependent manner. Besides, with the extension of the incubation time, a notable increase in IL-1β and LDH, as the main feature of pyroptosis (Shi et al., 2017), could be observed. These are consistent with the previous findings that pyroptosis mainly occurs in monocytes, macrophages, and also neutrophils (Wallach et al., 2016; Ryu et al., 2017) to induce lytic cell death, release bacteria, and expose them to reactive oxygen species (ROS) in neutrophils (Miao et al., 2010). These data, on the one hand, confirmed that a large number of GSDMD would be generated and secreted by NCs; on the other hand, after infection, secreted-GSDMD were released apace and also increased with time, which made it an ideal inflammatory marker. Moreover, the invariability of the level of intracellular GSDMD after 1 h may result in the formation of gasdermin pores and consistent outflow of cytoplasmic contents.

The pore in the cell membrane formed by the gasdermin-N domains will not only elicit pyroptosis, but also generate hyperactive cells, which possess lower amounts of GSDMD pores than pyroptotic cells (Evavold et al., 2018). During pyroptosis, the cell membrane is ruptured and cytosolic protein is released; the same goes with hyperactive cells. For instance, it has been confirmed that the pore-forming protein GSDMD regulates IL-1 release from hyperactive macrophages (Evavold et al., 2018). It hints that GSDMD may become a high sensitivity index than other inflammatory biomarkers. Compared with LDH and IFN-γ, also acting as inflammatory biomarkers (Liu et al., 2018; Tang et al., 2019), at the presence of *E. coli*, *S. aureus*, and *Salmonella*, GSDMD displayed the most rapid and largest degree of growth tendency of GSDMD in the culture supernatant. In the previous report, after 24 h, the concentration of IL-27 in the mononuclear cells culture supernatants at the presence of the Mtb antigen began to increase (Yang et al., 2012), but the concentration of GSDMD, which began to increase after 1 h, changed faster, and could make a contribution to an early diagnosis. These showed that GSDMD diagnosed infectious pneumonia with a higher sensitivity. Expressed by innate immune cells, IL-1β has been demonstrated to play a role in many inflammatory diseases, as well as in different cancers (Zhang et al., 2020). The higher diagnostic sensitivity at the presence of *P. aeruginosa* might lie in the following reason. The *Pseudomonas aeruginosa* mannose sensitive hemagglutination strain (PA-MSHA)-primed dendritic cells (DCs) directed T cell differentiation and primed Th cell to Th1 by an elevated secretion of IFN-γ (Zhang et al., 2014). In conclusion, under various kinds of pathologic status especially in the case of a bacterial infection, the level of GSDMD changed quickly and had a large variation in the amplitude, which improved the diagnostic sensitivity and the ability of an early diagnosis. Meanwhile, for regulating the release of the proinflammatory cytokines IL-1β and IL-18 and pyroptotic cell death, GSDMD would possess a higher diagnostic specificity. Besides, GSDMD had an advantage in diagnostic specificity in bacterial infections, which was the main cause of pyroptosis.

We further examined the change in PE-GSDMD during bacterial pneumonia treatment with an antibiotic. The responder was gradually decreased from the baseline during therapy, while non-responders showed the opposite tendency in PE-GSDMD. The research revealed that % of GSDMD change at day 4 could predict the treatment response at an early stage (AUC = 0.945, Specificity = 96.15%) and be better than other traditional indicators. The utility of a procalcitonin (PCT)-guided antibiotic treatment of intensive care patients has been postulated (de Jong et al., 2016). There is a research which reported that the AUC of PCT clearance rate at days 5 and 9 were 0.648 and 0.729, respectively, on the therapeutic effect of ventilator-associated pneumonia (Abula et al., 2014). As one of the important causes of toxic and side effects of some chemotherapy drugs, caspase-3 activation can trigger necrosis by cleaving GSDME (Wang et al., 2017b). This means that detection based on the GSDME expression level can be used as a better tool for prognosis interpretation. Besides, it is difficult to repeatedly extract PE from lung cancer patients. GSDMD is a small molecule protein that can be detected by chemiluminescence or immunoturbidimetric methods. Therefore, this new biomarker could be a routine quantitative testing by cost-effective analysis in clinical laboratories. However, there are some limitations to our study. Firstly, the limited population enrolled in this study may lead to bias. Also, it is difficult to rule out the misclassification of pleural fluid, especially the transudate group and PPE effusion, and the collection of PE is also an invasive test. Moreover, further study needs to be conducted to clarify the normal level of serum GSDMD.

## Conclusion

In brief, we found that during infections and some other diseases, a mass of GSDMD was secreted from NCs and released into PE rapidly. In addition, our study firstly manifests that pleural fluid GSDMD can be an effective novel biomarker for the early differential diagnosis of PE.

## Data Availability Statement

The raw data supporting the conclusions of this article will be made available by the authors, without undue reservation.

## Ethics Statement

The studies involving human participants were reviewed and approved by the Medical Ethics Committee of the Second Affiliated Hospital of Chongqing Medical University. The patients/participants provided their written informed consent to participate in this study.

## Author Contributions

PL and JS wrote the manuscript and conceived and designed the experiments. LD, BW, and LZo analyzed the data. LZa, JS, XZ, and YY collected and provided the sample for this study. WC fixed the manuscript. All authors have read and approved the final submitted manuscript.

## Conflict of Interest

The authors declare that the research was conducted in the absence of any commercial or financial relationships that could be construed as a potential conflict of interest.

## Abbreviations

GSDMD: gasdermin D
PE: pleural effusion
TPE: tuberculous pleural effusion
PPE: parapneumonic pleural effusion
MPE: malignant pleural effusion
ROC: receiver operating characteristic
NCs: nucleated cells
ADA: adenosine deaminase
LDH: lactate dehydrogenase
Mtb: *Mycobacterium tuberculosis*
NT-GSDMD: gasdermin-N domain
IL-1 β: interleukin-1 β
NSCLC: non-small cell lung cancer
NCCN: National Comprehensive Cancer Network
*E. coli*: *Escherichia coli*
*S. aureus*: *Staphylococcus aureus*
*P. aeruginosa*: *Pseudomonas aeruginosa*
LPS: lipopolysaccharide
PRR: pattern recognition receptors
PAMP: pathogen-associated molecular patterns
PA-MSHA: *Pseudomonas aeruginosa* mannose sensitive hemagglutination strain
DCs: dendritic cells
PCT: procalcitonin
PPV: positive predictive value
NPV: negative predictive value
LR+: positive likelihood ratio
LR-: negative likelihood ratio. The Cut-off value of GSDMD for TPE MPE, and PPE were 18.40 ng/mL 9.35 ng/mL, and 9.350 ng/mL respectively.
